# Supplementary material for: Hollow-tree super: A directional and scalable approach for feature importance in boosted tree models
Source: PLoS One. 2021 Oct 25;16(10):e0258658. doi: 10.1371/journal.pone.0258658 (PMC8544862; doi:10.1371/journal.pone.0258658)
Supplement: Attachment — Submitted filename: Response to reviewers [Hollow tree super].docx [file pone.0258658.s001.docx]

**Hollow-tree Super: a directional and scalable approach for feature importance in boosted tree models**

**Rebuttal Letter:**

Dear reviewers, thank you for firstly taking the time to review our manuscript, “Hollow-tree Super: a directional and scalable approach for feature importance in boosted tree models”, and secondly, for providing intricate feedback which we believe on the whole has improved the quality and scientific integrity of the manuscript. Below I have listed first the question asked by reviewers in *italics* with our subsequent responses and specific line numbers where changes have been made in blue and **bolded** text respectively. We look forward to hear your thoughts, and invite further revisions if the reviewers deem them necessary.

Kind regards,

Dr. Stephane Doyen (corresponding author)

**Reviewers' comments:**

*2. We note that one or more of the authors are employed by a commercial company: Omniscient Neurotechnology*

1. *Please provide an amended Funding Statement declaring this commercial affiliation, as well as a statement regarding the Role of Funders in your study. If the funding organization did not play a role in the study design, data collection and analysis, decision to publish, or preparation of the manuscript and only provided financial support in the form of authors' salaries and/or research materials, please review your statements relating to the author contributions, and ensure you have specifically and accurately indicated the role(s) that these authors had in your study. You can update author roles in the Author Contributions section of the online submission form.*

*Please also include the following statement within your amended Funding Statement.*

*“The funder provided support in the form of salaries for authors [insert relevant initials], but did not have any additional role in the study design, data collection and analysis, decision to publish, or preparation of the manuscript. The specific roles of these authors are articulated in the ‘author contributions’ section.”*

*If your commercial affiliation did play a role in your study, please state and explain this role within your updated Funding Statement.*

We thank the reviewers/editors for re-iterating their requirements for commercial entities submitting manuscripts. Indeed whilst all authors work for the company ‘Omniscient Neurotechnology’, the reviewers/editors are correct in their assumption that the only role the organization played in this manuscript's conception was author salaries. The author contributions listed in the original submission remain correct, and we will submit the statement provided in our amended funding statement in the page following our cover letter.

*2. Please also provide an updated Competing Interests Statement declaring this commercial affiliation along with any other relevant declarations relating to employment, consultancy,* ***patents****, products in development, or marketed products, etc.*

*Within your Competing Interests Statement, please confirm that this commercial affiliation does not alter your adherence to all PLOS ONE policies on sharing data and materials by including the following statement: "This does not alter our adherence to PLOS ONE policies on sharing data and materials.” (as detailed online in our guide for authors http://journals.plos.org/plosone/s/competing-interests) . If this adherence statement is not accurate and there are restrictions on sharing of data and/or materials, please state these. Please note that we cannot proceed with consideration of your article until this information has been declared.*

*Please include both an updated Funding Statement and Competing Interests Statement in your cover letter. We will change the online submission form on your behalf.*

We thank the reviewers/editors for re-iterating their requirements for competing interests when submitting manuscripts. We have since attached both the above funding statement, as well as an amended competing interest statement to our cover letter as specified in the above comment.

*Reviewer #1: 1. Kindly include a paragraph at the end of section 1 describing the flow and division of sections in the research paper.*

We thank their reviewer for their comment regarding summating the flow of information within the manuscript’s introduction. We have since expanded on the closing sentences of section 1 of the manuscript, highlighting the contents of the manuscript as they appear throughout the subsequent sections.

*2. The authors can add the formula for accuracy as an equation and explain the meaning of TP, TN, FP and FN in their problem.*

We thank the reviewer for their combined comment regarding providing formulas for metrics and further explanations for acronyms and abbreviations within the manuscript text. We have now added the formula for accuracy in line 300 of the methods, as well as explanations for TP (true positives), TN (true negatives), FP (false positives) and FN (false negatives) to lines 296-298, also within the methods.

*3. The authors have done few literature works, but they haven’t compared any of them in their experiment. I recommend the authors should compare their work with at least 2 works present in the literature.*

We thank the reviewers for their comments regarding broader appreciation and referencing of well-established literature in the field. We have made reference to a number of key manuscripts which utilize Gini, PDP and Permutation importance in sections 1.1.1, 1.1.2 and 1.1.3 of our manuscript which we believe bolster our arguments and provide further insight into the relative use cases of these traditional analyses.

*4. The authors should add few explanations why they have to choose GINI as there are lots of other feature selection algorithms such as IG, Chi, Pearson, and so on.*

We thank the reviewer for their comments regarding selections of GINI, PDP and Permutation importance in testing HOTS using the Iris dataset. Indeed these methods were selected based on our interpretation of the most commonly used algorithms in the field, but we agree that the fact that these were chosen over other common methods is an inherent flaw in our methodology. We have now added an additional limitation stating this fact, and whilst we still believe that these three methods are the optimal choices for visualisation and investigation within the manuscript body, we have conceded that assumptions shouldn’t be made that the concordance of results would translate between HOTS and IG, Chi-squared, and other feature importance methods in lines 499-505 of the limitations section.

*5. The authors can include a result analysis for each of the components (Gini Importance, Partial Dependence Plots, Permutation Importance)*

We thank the reviewer for their suggestion regarding including results for Gini, PDP’s and Permutation Importance within the results. As this manuscript presents HOTS as a new methodology independent to these three methods, and they are included insomuch as to test how closely HOTS reproduced the findings of them, we feel including separate sections in the results section for Gini, PDP’s and Permutation Importance would reduce the impact of the findings of HOTS, and take away from the core purpose of the paper which is to present this novel methodology in its own right. That being said, we have expanded on our methods section, further elaborating on the steps required to perform HOTS which can be found in lines 313-346 of the methods section.

*6. Please cite the following papers*

*1. Ashokkumar P, Siva Shankar G, Gautam Srivastava, Praveen Kumar Reddy Maddikunta, and Thippa Reddy Gadekallu. 2021. A Two-stage Text Feature Selection Algorithm for Improving Text Classification. ACM Trans. Asian Low-Resour. Lang. Inf. Process. 20, 3, Article 49 (April 2021), 19 pages. DOI:*[*https://doi.org/10.1145/3425781*](https://doi.org/10.1145/3425781)

*2. Maddikunta, P. K. R., Gadekallu, T. R., Al-Ahmari, A., & Abidi, M. H. (2020). Location based business recommendation using spatial demand. Sustainability, 12(10), 4124.*

We thank the reviewer for their desire to broaden the spectrum articles supporting our claims. We have carefully read the above mentioned manuscripts and have found:

- The first paper appears to present a feature selection algorithm which optimizes text recognition by reducing the number of entered features within a model through filtering.
- The second paper presents an algorithm which uses keyword searches in order to suggest the optimal location and content for new businesses in high-competition areas.

Whilst both of these papers suggest interesting methods for achieving their outcomes, we, the authors have failed to identify a way to integrate them in support of the arguments made within this manuscript. We nevertheless remain open to the opinions of the reviewers as to how they think they would fit into the manuscript should they believe them necessary in support of any arguments made. We have however made reference to a number of additional works which can be found within the references section of the manuscript re-submitted.

*Reviewer #2: 1. The paper is written in a good manner. Some minor touches can improve this paper more.*

*2. The quality of the figures can be improved more.*

We thank the reviewer for their suggestions regarding the presentation of figures within the manuscript. We have made substantial adjustments and improvements to figures 1,6, and 7 of the manuscript, as well as have made the decision to convert figure 4 into a table, which we feel is a more easily-interpretable medium for visualising the relative feature importances across the five folds presented. We hope this satisfies the reviewers concerns, yet are open to continued criticism and adjustment of these figures at the reviewers discretion.

*3. The contributions of the authors are not clear. They have mentioned in first contribution.*

We thank the reviewer for their comment and apologise for any confusion caused by the author contribution list - in accordance with the PLOS styling guidelines, we have now added symbols to the author list presented on page 1 of the manuscript to reflect senior and contributing authors, who contributed relatively similar contributions to this manuscript. Within our submission we have also re-iterated and ensured the check boxes for contributions accurately reflect the relative roles the authors played in the construction of this manuscript.

*4. Future research directions section is core, however, it is not good at all.*

We thank the reviewer for their suggestions on the future directions of the results, and are in agreement that we should elaborate with excitement the potential for HOTS’ use in future investigations. We have added additional future directions relating to our chosen field of neuroscience that can be found in lines 529-537 of the manuscript.

*5. What are the computational resources reported in the state of the art for the same purpose?*

*- Please cite each equation and clearly explain its terms.*

*- Clearly highlight the terms used in the algorithm and explain them in the text.*

We apologize for any confusion our original manuscript presented with insufficient explanation of equations and terms. We have now added term definitions as well as an equation for accuracy which can be found in the methods section of the manuscript.

*6. What are the evaluations used for the verification of results?*

We thank the reviewer for their enquiry into the evaluations used for the verification of results. Our method of verifying the results on the iris dataset was to compare the top features as determined by HOTS with those identified using the other feature importance methods (Gini, PDP and Permutation importance) - whereby the overlap here served as verification. Proper verification of the results on the Schizconnect Cobre dataset would require treatment on the HOTS identified regions and a followup symptom assessment which was not possible given the dataset did not contain “pre” and “post” imaging data. A less formal verification was performed by investigating the top features identified which showed to be relevant to the disease being studied. We have noted in the future directions that the reviewers recommendations of verifying the neuroscientific results would be a worthwhile exercise.

*7. Several paragraphs contain trivial information and should be dropped.*

We thank the reviewer for their comment regarding streamlining the information presented within the manuscript - we have made ongoing adjustments to the manuscript body paragraphs, and removed sections which we deemed to be trivial information.

*8. I found some English mistakes please check them.*

We thank the reviewer for their comment regarding the syntax and spelling of the manuscript - we have now checked the information of the manuscript and have removed all mistakes within it.

*9. Kindly refer the below paper:*

*1. Rajput, D.S., Basha, S.M., Xin, Q. et al. Providing diagnosis on diabetes using cloud computing environment to the people living in rural areas of India. J Ambient Intell Human Comput (2021).* [*https://doi.org/10.1007/s12652-021-03154-4*](https://doi.org/10.1007/s12652-021-03154-4)

We thank the reviewer for their desire to broaden the spectrum articles supporting our claims. We have carefully read the above mentioned manuscripts and have found:

This manuscript presents an algorithm for optimizing patient-doctor interactions in those suffering from diabetes in rural areas through disseminating necessary information and warning of risk factors for those in the early stages / unaware of their condition.

Whilst both of these papers suggest interesting methods for achieving their outcomes, we, the authors have failed to identify a way to integrate them in support of the arguments made within this manuscript. We nevertheless remain open to the opinions of the reviewers as to how they think they would fit into the manuscript should they believe them necessary in support of any arguments made. We have however made reference to a number of additional works which can be found within the references section of the manuscript re-submitted.
